# Translational Approach to Induce and Evaluate Verocytotoxic *E. coli* O138 Based Disease in Piglets

**DOI:** 10.3390/ani11082415

**Published:** 2021-08-17

**Authors:** Luciana Rossi, Lauretta Turin, Giovanni Loris Alborali, Eugenio Demartini, Joel Fernando Soares Filipe, Federica Riva, Pietro Riccaboni, Eugenio Scanziani, Paolo Trevisi, Paola Dall’Ara, Matteo Dell’Anno, Antonella Baldi

**Affiliations:** 1 Department of Health, Animal Science and Food Safety “Carlo Cantoni” (VESPA), Università degli Studi di Milano, 26900 Lodi, Italy; luciana.rossi@unimi.it (L.R.); eugenio.demartini@unimi.it (E.D.); matteo.dellanno@unimi.it (M.D.); antonella.baldi@unimi.it (A.B.); 2 Department of Veterinary Medicine, Università degli Studi di Milano, 26900 Lodi, Italy; joel.soares@unimi.it (J.F.S.F.); federica.riva@unimi.it (F.R.); pietro.riccaboni@unimi.it (P.R.); eugenio.scanziani@unimi.it (E.S.); paola.dallara@unimi.it (P.D.); 3 Lombardy and Emilia Romagna Experimental Zootechnic Institute (IZSLER), 25124 Brescia, Italy; giovanni.alborali@izsler.it; 4 Department of Agricultural and Food Sciences, Università di Bologna, 40126 Bologna, Italy; paolo.trevisi@unibo.it

**Keywords:** *Escherichia coli*, O138, verotoxin, VTEC, pig, infection, post weaning diarrhoea, oedema disease, immunity, experimental infection

## Abstract

**Simple Summary:**

The aim of the study was to set up experimental conditions to simulate the simultaneous outbreak of post-weaning diarrhea and enterotoxaemia in weaned piglets, through verocytotoxic O138 *Escherichia coli* challenge. Zootechnical, clinical, microbiological, histological and immunological parameters were evaluated along the follow-up of control and infected groups. Results showed that experimental infection significantly affected the clinical status. Infected animals showed significant higher total median scores of epiphora, vitality, hair irregularity, oedema and depression; in addition, they displayed evident inflammatory infiltrate of lymphocytes, and follicular hyperplasia, increase of IgG in the intestinal crypts and CD3-positive T cells in intestinal epithelium. The infection model, carried out on receptor-mediated susceptible piglets, allowed to identify a discriminative panel of clinical symptoms related to *Escherichia coli* O138 infection and could be used to assess the protective effect of antibiotic alternatives.

**Abstract:**

Pig livestock was influenced by several global concerns that imposed a re-thinking of the farming system, which included the reduction in chemical dependency and the development of antimicrobial alternatives. Post-weaning diarrhea and enterotoxaemia caused by *Escherichia coli,* are serious threats that are responsible for the economic losses related to mortality, morbidity and stunted growth in weaning piglets. The aim of the study was to set up experimental conditions to simulate the simultaneous outbreak of post-weaning diarrhea and enterotoxaemia in weaned piglets, through verocytotoxic O138 *Escherichia coli* challenge, with a multidisciplinary approach. Eighteen piglets susceptible to F18 VTEC infection were selected by polymerase chain reaction for polymorphism on the fucosyltransferase 1 gene and randomly divided in two experimental groups, non-infected controls (C; *n* = 6) and infected ones (I; *n* = 12) and housed into individual pens at the same environmental conditions for 29 days. At day 20, I pigs were orally inoculated with *Escherichia coli* O138 and fed a high protein ration for 3 days. Zootechnical, clinical, microbiological, histological and immunological parameters were evaluated along the follow up (3 and 9 days). Experimental infection, confirmed by bacteria faecal shedding of the I group, significantly affected the clinical status. The I group showed significantly higher total scores, corresponding to medians of the sum of daily scores from days 1 to 3 (Σ3) and 1 to 9 (Σ9) post infection, epiphora, vitality, hair irregularity, oedema and depression. Histological examination showed evident inflammatory infiltrate of lymphocytes, and follicular hyperplasia in I pigs; in the same group, the immunohistochemical and immunological assays revealed an increase in IgG in the intestinal crypts and CD3-positive T cells in intestinal epithelium. The experimental *Escherichia coli* infection in controlled conditions is crucial for both the evaluation of innovative compounds and the elucidation of the mechanisms associated with the persistence of antibacterial resistant strains. In conclusion, the adopted infection model, carried out on receptor-mediated susceptible piglets, allowed us to identify a discriminative panel of clinical symptoms related to *Escherichia coli* O138 infection, and could be used to assess the protective effect of antibiotic alternatives.

## 1. Introduction

Improving the sustainability of pig livestock is a priority of global policies, in line with the agroecology principles, that imposed a re-thinking of the farming system, including the reduction in chemical dependency and the development of antimicrobial alternatives [1,2,3,4].

The most critical phase of pig production is weaning, characterized by gastrointestinal disorders, such as post-weaning diarrhea (PWD), mainly due to *Escherichia coli* O138, O139, and O141 serogroups, which causes important economic losses (mortality, morbidity and stunted growth) [5,6]. Two important bacterial virulence factors are F18 adhesive fimbriae and verocytoxin (VT2e), often found in association in the β-haemolytic O138 *E. coli* strain [7,8]. The fimbriae are responsible for the attachment to porcine intestinal villi, while the toxin destroys endothelial cells of small vessels, resulting in blood clots, hemorrhage, ischemic necrosis, and oedema in vital organs, including the brain [9,10,11,12,13].

After the European ban of antibiotics [14], traditionally used to control PWD, an increased use of Zinc oxide (ZnO) was observed to control enteric disorders, with consequent negative impacts on the environment and enhanced spread of antibiotic-resistant strains [15,16,17]. Therefore, the development of effective alternative tools is needed to reduce disease incidence and guarantee the performance, health and welfare of pigs. A number of innovative replacements have been thoroughly discussed, such as edible vaccines, immunotherapeutics, bacteriophages, antimicrobial peptides, gut microbiota modulators, plant extracts, and integrated approaches [18,19,20,21].

PWD is a multifactorial disease in which the interaction of host-infectious agent, environment, management strategies, age and genetics and immunologic factors play critical roles in the outcome [22,23]. Therefore, a more comprehensive understanding of these aspects is needed, and is achievable through the experimental reproduction of the disease in controlled and repeatable conditions. Although knowledge has advanced substantially in recent decades, much more needs to be learned about the pathogenesis and the control of PWD outbreaks, which are increasingly spreading worldwide.

The aim of the study was to set up experimental conditions to simulate the simultaneous outbreak of PWD and enterotoxaemia in weaned piglets, through verocytotoxic O138 *Escherichia coli* (F18 positive) challenge and apply a multidisciplinary investigative approach. In particular, the animals were evaluated for nutritional traits, zootechnical performance, clinical signs, immunohistochemical and immunological parameters.

## 2. Materials and Methods

### 2.1. Animal Housing and Experimental Challenge

The animals susceptible to verocytotoxic O138 *E. coli* were selected by polymerase chain reaction for the polymorphism on α 1,2 fucosyltransferase (FUT1) gene [24,25]. Genomic DNA of each pig was extracted from duodenum tissue using the Wizard^®^ Genomic DNA Purification Kit (Promega Italia S.r.l, Milan, Italy).

PCR reaction for the FUT1 gene was performed using 0.3 μM of each specific primer (F: CTTCCTGAACGTCTATCAAGACC; R: CTTCAGCCAGGGCTCCTTTAAG) in a volume of 20 μL containing 2 μL of 10× standard buffer, 0.75 mM MgCl_2_, 160 μM dNTPs, 1 U Taq polymerase (Fisher Molecular Biology, Rome, Italy), and 1 μL containing 50–100 ng template DNA according to the following steps: denaturation at 95 °C for 3 min, 30 cycles of denaturation at 95 °C for 30 s, annealing at 56 °C for 30 s, and extension at 72 °C for 30 s in a thermocycler (Applied Biosystems 2720, Waltham, MA, USA) [26]. The FUT1 amplicon underwent restriction analysis with 7 U HhaI (Thermo Fisher Scientific, Waltham, MA, USA) in a final volume of 25 µL overnight at 37 °C, and electrophoresis in 10% polyacrylamide gel for visualization of the restriction fragments by GelRed (Olerup SSP AB, Stockholm, Sweden).

Piglets (Large White × Landrace) aged 22 ± 2 weaned at 21 ± 2 days from a conventional farm free from diseases, without history of verocytotoxic *E. coli* infections and negative for the presence of haemolytic *E. coli* in faeces, were transported to the Experimental Animal Research and Application Centre of the University of Milan in Lodi (Italy), allocated in individual pens (100 × 50 cm) and maintained under controlled conditions (27–29 °C; relative humidity 60%), with plastic slatted floor, nipple drinker, environmentally safe eco and easily chewable enrichment material; water and feed were administered ad libitum. This in vivo trial complied with Italian law on animal experimentation and ethics (Italian Health Ministry authorization number 102/2015-PR) in accordance with European regulation [27].

The animals, checked daily for health status, were divided in two experimental groups: non-infected controls (C; *n* = 6) and infected ones (I; *n* = 12). The infection was performed at day 20 post arrival with verocytotoxic *E. coli* O138 strain previously tested positive for F18 and Vt2eB genes [28]. One hour before infection, piglets were sedated intramuscularly with azaperon (2 mL/each, Stresnil^TM^, Janssen Cilag SpA, Milan, Italy), thereafter 30 mL of 10% bicarbonate solution (Sigma-Aldrich, Saint Louis, MO, USA) was orally administered [29]. After 10–15 min, the bacteria inoculum was given (5 mL containing 10^10^ O138 F18 *E. coli* CFU) through intragastric catheter according to Rossi et al. [19].

The piglets were fed a basal diet with zinc content 50 ppm, without antibiotics (Ferraroni S.p.A, Bonemerse, Italy), except for three days after the infection, when they received a high protein content diet (25.42 g/kg dry matter) (Table 1).

### 2.2. Zootechnical Evaluation and Sample Collection

The rectal temperature was measured daily along with the faecal consistency, which was recorded according to a four-level scale: 0 = normal (faeces firm and well formed), 1 = soft consistency (faeces soft and formed), 2 = mild diarrhoea (loose faeces, usually yellowish), 3 = severe diarrhoea (faeces watery and projectile).

Faecal samples were collected from rectum every day after the infection for microbiological assays and immunoglobulin titration.

The typical clinical signs of VTEC infection (palpebral oedema, epiphora, vitality, depression, hair, perineal area, respiratory and neurological problems) were registered daily by the same veterinary surgeon, according to a three-level points scale score described by Rossi et al. [19]. Palpebral oedema is defined as a pathological condition (related to the vasopermeabilization caused by verocitotoxins) characterized by the accumulation of fluid in the tissues of the inner part of the eyelids with different degree of severity: 0 = normal; 1 = mild bilateral oedema (puffy eyelids giving a sleepy appearance); 2 = severe (prominent and closed eyelids). Epiphora is an overflow of tears due to the inflammation and the reduced motility of eyelid or of the lacrimal pump. It was classified with the following score: 0 = normal; 1 = mild (moderate presence of eye discharge material in the corner of the eye); 2 = severe (abundant presence of brown discharge material in the corner of the eye); for the vitality and depression scores a disturbed behaviour was defined as slow reactions, an unsteady and slow gait whilst walking and an inattentive response when encouraged to move. Vitality score: 0 = good; 1 = loose (failure to react to stimulus); 2 = bad (slow response to stimuli); depression score: 0 = normal status; 1 = mild (slow reactions); 2 = high (lethargy); perineal area: 0 = clean; 1 = smear; 3 = smear with flogosis (red area caused by inflammation); respiratory score: 0 = normal; 1 = slightly quick respiratory rate; 2 = quick respiratory rate with open mouth; neurological score: 0 = normal; 1 = mild symptoms (incoordination); 2 = severe symptoms (lateral position, paddling limbs, central nervous symptoms); hair condition was evaluated on the dorsal of porcine skin. Hair score: 0 = regular (smooth, clean, flat and uniform); 1 = slightly irregular (fuzzy hair coat and/or scaly skin); 2 = irregular (bald patches, or a rough, dull uneven coat and reddened skin).

All piglets were individually weighed on day 0, 7, 13, 20, 22, 27 and 29 post infection while the feed intake (FI) was daily measured weighing the residual feed at the pen level (experimental unit for FI evaluation). Then, individual average daily gain (ADG) and gain to feed ratio (G:F) were calculated.

Blood was collected from the jugular vein of each animal on days 20, 27 and 29 to determine microhematocrit and serum antibodies. Two animals of C (C3) and four animals of I (I3) groups were sacrificed at day 3 post infection and all the others were euthanized at day 29 (C9/I9). Samples (approximately 0.5 cm^3^) of jejunum, mesenteric lymph nodes and intestinal scrapes were obtained immediately after euthanasia and placed in RNA Later (Qiagen, Germany) for gene expression analysis; another part of the same excised samples was frozen for immunochemistry, and another one was fixed in 10% neutral buffered formalin for immunohistochemistry/histology. Samples of the ileum (one for each euthanized animal) were collected immediately after the euthanasia and carefully sealed with two sterile cotton ligatures, 3 cm apart from the proximal to the distal one and carried to the microbiology laboratory at 4 °C for microbiological analyses.

### 2.3. Microbiological Analysis

Faecal samples were analysed for the animal selection and the confirmation of experimental infection procedures. Briefly, faeces (1 g/animal) homogenized in 1 mL of sterile saline buffer were plated onto 5% sheep blood agar plates (Blood Agar Base No. 2, Oxoid, Hampshire, UK) and incubated overnight at 37 °C to evaluate the presence of hemolytic colonies. Up to 5 hemolytic colonies were selected from each plate and grown on MacConkey agar (Oxoid, Hampshire, UK), Triple Sugar Iron agar (Oxoid, Hampshire, UK), Simmons Citrate agar (Oxoid, Hampshire, UK), and Buffered Peptone Water Broth (Oxoid, Hampshire, UK). Colonies positive for glucose oxidation–fermentation, fermentation of lactose (MacConkey agar), indole production (peptone water + reagent Kovacs—Kovac’s reagent for indoles—Fluka, Saint Louis, MO, USA) and sodium citrate-negative were then subjected to biochemical tests with the API system^®^ (API 20 NE—BioMerieux, Marcy-l’Étoile, France) for a more precise identification. The identification of the *Lactobacillus* spp. and *Enterobacteriaceae* was performed on plates specific for each type of bacteria and incubated under adequate conditions.

A semi-quantitative approach, based on serial dilutions in medium allowed for the determination of the bacteria concentration (detection limit 1 CFU/g).

*E. coli* strains were isolated from faecal samples of each pig, cultured (37 °C for 24 h) on Blood Agar and MacConkey agar (Oxoid, Hampshire, UK), further isolated (37 °C for 24 h) on Trypticase Soy agar (Oxoid, Hampshire, UK) and biochemically identified with API-20E method (BioMerieux, Marcy-l’Étoile, France). Besides the evaluation of hemolytic activity, they were serotyped by using monospecific antisera for the somatic antigen O138 [30]. Finally, the genetic characterization was performed by PCR on isolated *E. coli* strains for VT, VT1, VT2 and VT2e; the VT2e positive strains were also tested for F18 [31,32].

### 2.4. Histopathology and Immunohistochemistry

The organs fixed in 10% neutral buffered formalin were paraffin wax embedded, sliced with a microtome into five μm thick histological sections and stained with hematoxylin and eosin. Samples were examined for presence of inflammation both in villi and in *lamina propria* (infiltrates of lymphocytes, plasma cells, histiocytes and eosinophils), epithelial regeneration (enterocytes with high nucleus/cytoplasm ratio on the intestinal layer), fusion of villi, oedema in deep *lamina propria*, and T atrophy (T-dependent ipocellularity areas), stroma (fibroconnective and histiocytes) and follicular hyperplasia. All histological parameters were semi-quantitatively scored: 0 = absent, 1 = slight, 2 = moderate; 3 = strong.

Sections of formalin-fixed paraffin-embedded tissues were dewaxed and unmasked by heat induced epitope retrieval and Buffer H (Bio-Optica, Milan, Italy); endogenous peroxidase activity was blocked by incubation with 3% H_2_O_2_. After rinsing and treatment with PBS, containing normal serum to reduce nonspecific background staining, sections were incubated with the primary antibodies specific for Iba1 (Wako, Neuss, Germany), CD3 (Dako, Næstved, Denmark), CD20 (Thermo Scientific, Rome, Italy), IgG (Vector Laboratories, Burlingame, CA, USA) and IgA (Abcam, Cambridge, UK) at the suggested dilutions. Sections were then incubated with biotinylated secondary antibody (rabbit anti-goat, Vector Laboratories, Burlingame, CA, USA) and labelled by the avidin-biotin-peroxidase (ABC) procedure (commercial immunoperoxidase kit VECTASTAIN^®^ Elite ABC-Peroxidase Kit Standard, Vector Laboratories, Burlingame, CA, USA). The immunoreaction was visualized with 3,3′-diaminobenzidine substrate (Peroxidase DAB Substrate Kit, Vector Laboratories, Burlingame, CA, USA) after counterstaining with Mayer’s haematoxylin (Merck, Kenilworth, NJ, USA) [33] and blind evaluated with a light microscopy by a veterinary pathologist. Known positive control sections were included in each assay. CD3, CD20 and Iba1 immunoreactions were semi-quantitatively scored in *lamina propria* (0 = absent, 1 = rare cells, 2 = some cells; 3 = numerous cells; 4 = very numerous cells), while IgG and IgA immunoreaction was semi-quantitatively scored on the epithelial luminal surface and *lamina propria* (0 = absent, 1 = slight, 2 = moderate; 3 = strong).

### 2.5. Immunoenzmatic Assay

Intestinal scrapes were homogenized using a rotor-stator system (Ultra Turrax T25, Staufen im Breisgau, Germany) in lysis buffer (50 mM Tris–HCl, pH 7.4, 1% Nonidet P-40, 0.25% sodium deoxycholate, 150 mM NaCl, 1 mM EDTA, Sigma-Aldrich, Saint Louis, MO, USA) with protease inhibitor cocktail (1 mM PMSF, 5 μg/mL Complete Protease Inhibitor Cocktail, Roche diagnostics, Mannheim, Germany) and centrifuged at 470× *g* for 15 min at 4 °C. The supernatants were collected and the protein content was quantified by direct absorbance measurement at 280 nm in quartz cuvette.

The titers of serum and intestinal mucosa total IgA (as mg/mL and ug/mL, respectively) were determined using a sandwich ELISA (Swine IgA ELISA Quantitation Set, Bethyl Laboratories Inc., Montgomery, TX, USA) according to the manufacturer’s instructions. Intestinal mucosa TNF-α, IL-8 and CXCL9 (MIG) levels (ng/mg of total proteins) were detected using three specific Swine Do-it-Yourself sandwich ELISA kits (Kingfisher Biotech Inc., St. Paul, MN, USA), while intestinal mucosa IL-1β was determined using Porcine Interleukin 1β ELISA kit (Cusabio Life Science, Houston, TX, USA). Negative and blank (buffer) samples were included on each plate along with duplicates of samples.

### 2.6. RNA Extraction, Reverse Transcription and Real-Time PCR Assays

Total RNA was isolated from the samples homogenized by a rotor-stator system (Ultra Turrax T25, Staufen im Breisgau, Germany) in 2 mL of TRI^®^Reagent (Sigma-Aldrich, Saint Louis, MO, USA). Purity and concentration of total RNA were spectrophotometrically determined (BioPhotometer Eppendorf, Hamburg, Germany) and 1 µg of total RNA from each sample was reverse transcribed to cDNA in a 25 µL final volume using the High-Capacity cDNA Archive kit (10 min at 25 °C, 60 min at 37 °C and 5 min at 95 °C) (Applied Biosystems, Waltham, MA, USA) [34]. The resulting cDNA was assayed in Real-Time PCR (7000 Sequence Detection System, Applied Biosystem, Waltham, MA, USA) [35] to quantify the expression of the best-characterized innate immunity receptors TLRs (2 and 4) in swine and pro-inflammatory cytokines IFN-γ and IL-1β in jejunum, and the expression of the antigen-presenting molecules MHC (type I and II) in mesenteric lymph node cells. Specific primer pairs were designed on the NCBI nucleotide sequences database. The porcine housekeeping gene beta-actin was used as endogenous control. All the primers were custom synthesized (Invitrogen, Waltham, MA, USA) (Table 2).

Gene expression of each sample was quantified in relationship to an animal chosen as calibrator (relative quantification) and normalized using the calculated beta-actin cDNA expression (mean) of the same sample and run [34] and results were reported as relative expression compared to beta-actin. 

### 2.7. Statistical Analysis

The results were analysed using the Wilcoxon Rank Sum test for unpaired samples using JMP^®^ Pro 15 (SAS Inst. Inc., Cary, NC, USA). The statistical differences were considered when *p* ≤ 0.05.

Differences were first tested between C and I animals. Furthermore, the differences between 3-day and 9-day sacrificed animals were verified within C and I groups, respectively. Finally, the differences between C and I animals within 3-day and 9-day sacrificed animal groups were also tested. All measurements were assessed using the individual pig as an experimental unit. The results are presented as medians and range (minimum–maximum).

## 3. Results

### 3.1. Zootechnical and Clinical Evaluation

The polymorphism analyses on the FUT1 gene proved that all piglets used in this study were susceptible to F18 VTEC infection. The digestion of amplified FUT1 resulted in fragments of 241, 93 and 87 bp for the FUT1G/G genotype, whereas the FUT1G/A genotype generated fragments of 328, 241, 93 and 87 bp and the FUT1A/A genotype created fragments of 328 and 93 bp.

The uninfected animals maintained the average daily rectal temperature below 39 °C for all the time course of the study, while the infected piglets showed an increase of about half a degree. The evaluation of the clinical status (faecal consistency, palpebral oedema, epiphora, vitality, depression, hair, perineal area, respiratory and neurological problems) is reported in Table 3 as median score, corresponding to the sum of individual daily scores from days 1 to 3 (Σ3) and 1 to 9 (Σ9) post infection. In general, all uninfected animals showed lower values for all the measured clinical signs. The infection induced statistically significant higher scores for epiphora, oedema, vitality and depression in the first 3 days and for oedema, depression, vitality and hair at day 9 post infection.

**Figure 1:**
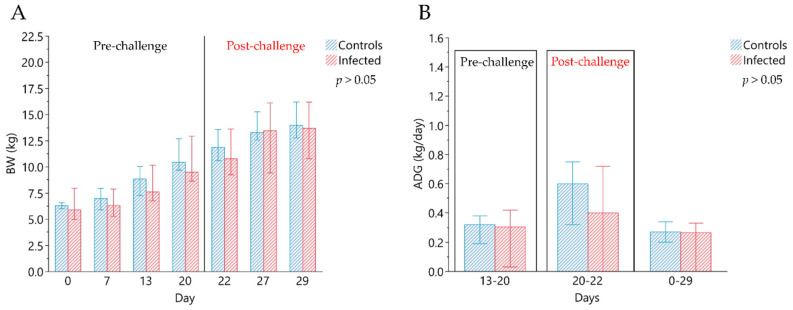
Evaluation of zootechnical performance. (**A**) Median values of individual body weight (kg) of experimental groups, measured at 0, 7, 13, 20, 27 and 29 days. Day 0 corresponds to the beginning of the in vivo trial, and day 20, indicated by the line, correspond to the day of infection. (**B**) Median values of average individual daily gain (ADG) in the experimental groups. ADG (13–20) indicates the average daily gain calculated in the pre-infection period: from day 13 to day 20; ADG (20–22) represents the average daily gain in the first period after infection (from day 20 to day 22; at day 20 was performed the experimental infection); ADG (0–29) is the average daily gain calculated in the entire experimental period (from day 0 to day 29). The comparisons were performed using the Wilcoxon Rank Sum test for independent groups. All data are reported as medians, minimum (min) and maximum (max) of results obtained for the pigs in the Control (*n* = 6) and in the Infected (*n* = 12) groups.

Although the median BW of the C group was higher than the I group, significant differences were not detected (Figure 1). The median ADG in the pre-infection period (days 13–20) was 315.71 g/day (min 191.43–max 380) and 305.72 g/day (min 28.57–max 422.86) in C and I animals, respectively. Two days after the infection (ADG 20–22) median values observed in C and I groups showed an ADG of 597.50 g/day (min 320.00–max 750.00) and 402.50 g/day (min 190.00–max 715.00), respectively. The I group showed a median value of average individual FI of 331 g/day (min 210–max 426) and 562 g/day (min 431–max 731), respectively, in the pre-infection period (day 0–20) and in post infection period (21–29 days). The average daily FI of C pigs was 382 g/day (min 263–max 434) and 570 g/day (min 460–max 717), calculated on days 0–20 and on day 21–29, respectively; although slightly higher, did not show significant differences. No differences were observed for the feed efficiency in the pre-infection period (G:F, 0.60, min 0.47–max 0.78 and 0.63, min 0.08–max 0.74 in C and I groups, respectively), while in the post infection period a higher feed efficiency was observed for C group (G:F, 0.71, min 0.67–max 0.71 in C vs. 0.59, min 0.54–max 0.69 in I group; *p* < 0.01).

### 3.2. Microbiological Analysis

**Figure 2:**
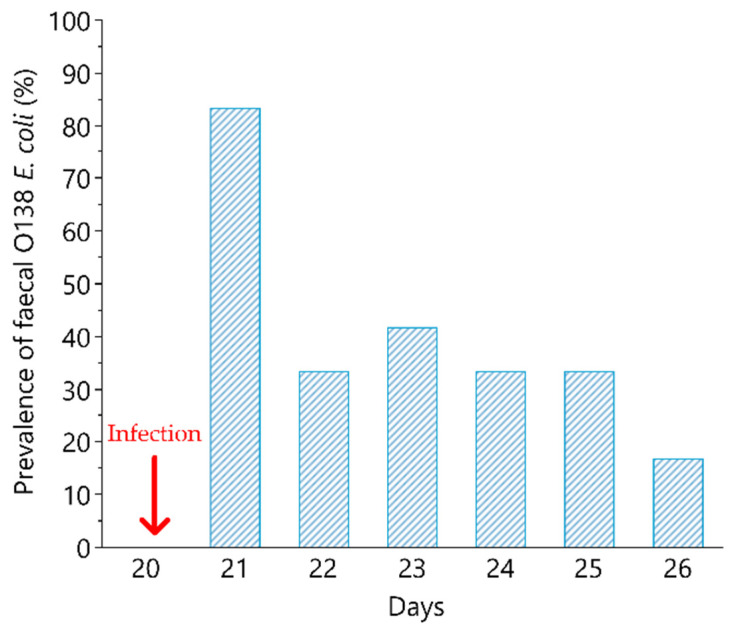
O138 F18 *E. coli* shedding after experimental pig infection. Percentage of piglets of the infected group (*n* = 12) presenting O138 F18 *E. coli* in faeces in the post infection period (day 21 to day 28). The experimental infection was represented by a single intragastric inoculum (5 mL) equivalent to 1 × 10^10^ O138 F18 *E. coli* colony-forming units (CFU)/5 mL and was performed at day 20. The arrow indicates the day of infection.

None of the piglets presented faecal shedding of O138 and F18 *E. coli* strains before infection, neither did C animals over the time course of the study. Faecal shedding of hemolytic O138 and F18 *E. coli* was detected in 83% (*n* = 10) of I group the day after the infection (Figure 2).

Only 1 piglet of the I group did not shed *E. coli* O138 in the observed period (1 to 7 days after infection). The average duration of faecal shedding, in the 8 days considered after infection, was 2.5 days (ranging from 1 to 7).

The total faecal bacterial count decreased gradually from the day after challenge, returning after 7 days at the same level of the pre-infection period. The total bacterial count ranged from 6.6 to 10.4 CFU/L in the experimental group.

### 3.3. Histology and Immunohistochemistry

The only statistically significant value detected by histology was the inflammatory infiltrate of lymphocytes, which was higher in I vs. C piglets (higher values detected at day 9 post infection). Infiltrates of lymphocytes significantly decreased from 3 to 9 days post-infection in the C group (*p* < 0.05). The immunohistochemistry results showed statistically significant higher CD3-positive T cells in intestinal epithelium in C vs. I animals sacrificed at day 3, and statistically significant lower cells in the animals euthanatized at day 9 (*p* < 0.05) (Table 4). Non-infected pigs displayed a significant decrease in CD3-positive T lymphocytes in epithelium and *lamina propria* from day 3 to 9 (Table 4). When considering only infected piglets both the CD3-positive T cells in epithelium and IgG in the intestinal crypts resulted statistically significantly higher in the subacute phase vs. the acute one (Table 5).

### 3.4. Immune Parameters

Within the C group, the serum titer of IgA, TNF-α and CXCL9 tended to decrease from 3 to 9 days. IgA measured in the small intestinal scrapes and IL-8 increased from day 3 to 9 post infection although without a statistical significance (Table 6). Within the I group, no statistical differences were observed between days 3 and 9 post infection (Table 7).

Comparing C and I groups at day 3 post infection, no significant differences were observed (Table 7), while at day 9 post infection IgA in the small intestinal scrapes tended to be lower in I vs. C. None of the parameters measured by real-time RT-PCR showed significant differences.

## 4. Discussion

An appropriate disease model is necessary to study the pathogenesis of the multifactorial PWD and to develop control strategies alternative to the use of antibiotics. The disease depends not only on virulence or pathogen–host interaction, but also on pig resistance and immune response. In this experimental model, in order to reduce the number of animals, piglets were chosen after polymorphism analysis of the FUT1 gene, correlated with F18 receptor expression [36], susceptible to infection with *E. coli* O138.

In order to bypass the first line of body’s defence against the invading microorganism, prior to experimental infection, piglets received a solution of bicarbonate, which reduces the neutralization of ingested bacteria by gastric acids.

The high-CP diet administered during the first three days after the infection was intended to be a “high-risk” diet, since several pathogens preferentially ferment proteins and high-CP diet in newly weaned piglets is indicated as one of the predisposing factors of VTEC infection. Moreover, the high-CP was due to soybean, which seems to favour the occurrence of *E. coli* infection [19].

The occurrence of VTEC infection was confirmed by bacteria faecal shedding in all the I group piglets and none of the C group ones. It peaked at day one post infection for the majority of animals and subsequently decreased in the following 7 days. A broad spectrum of clinical outcomes, including diarrhoea and enterotoxiaemia, which typically do not occur in all infected piglets, was also observed.

Zootechnical performances were impacted after challenge. The ADG reduction (days 20–22) was linearly related to the FI reduction in I group. Despite this significant reduction, the final BW was not statistically different in the two groups, probably due to compensatory FI starting 4 days after challenge, normally observed in *E. coli* infection. The feed efficiency was lower in the infected animals; the total recovery may take far longer than this experimental period.

The clinical symptoms of *E. coli* O138 infections are mainly related to verotoxins and F18 adhesins, which damage the vascular endothelium of the small intestine, subcutis, and brain and ultimately lead to subcutaneous oedema and neurological disorders. The animals showed, after infection, a broad spectrum of clinical outcomes, including specific (palpebral oedema and epiphora) and aspecific (depression, reduction in vitality) symptoms. In particular, the medians of sum of the daily scores of palpebral oedema, epiphora and vitality resulted significantly higher in the infected group; this variation could be used to monitor the infection in the acute phase (Σ3). A higher depression level and a reduction in the vitality were also measured in infected animals, both in acute and subacute phases, highlighting the importance of considering the role of behavioural alteration on disease outbreaks that often contribute to a reluctance to move.

The faecal scores were higher in infected animals, but not statistically significant. This may be explained with the limited number of animals enrolled in this study and possibly also for the increased intestinal fermentation due to high-CP diet administered to both experimental groups. The effect of the infection on the hair score was observed 9 days post bacterial inoculation (hairs appeared normal in the first few days). The development of fuzzy hair coat, lumps, scaly skin and bald patches is typically used in field as a parameter to identify compromised pigs and/or to evaluate their welfare. It is not considered a specific symptom of stress reaction that can be considered in the general evaluation of health status. The absence of mortality may depend on the adoption of a unique infection model, in line with the 3Rs guiding principles for more ethical use of animals in testing, and on environmental condition. The group housing of the animals contributes to increase the spread of the bacteria in the environment and the transmission.

Considering the immune parameters, proinflammatory cytokines and chemokines, such as TNF-α, IL-1β, IL-8, CXCL9 and IFN-γ, directly or indirectly mediate inflammatory response and are essential in initiating effective protection [37]. Pig weaning has been reported to upregulate the expression of inflammatory cytokines in the gut during acute stress [38]. The decrease in cytokines and chemokines (TNF-α and CXCL9) from day 3 to 9, observed in C group, may be explained with a diminished acute stress. In humans, it is known that the proinflammatory cytokine production increases with acute stress and decreases with chronic stress.

Immunohistochemistry confirmed the increase in inflammatory parameters in all infected animals.

Once the invading pathogen enters the host through mucous membranes, local rather than systemic immunity is essential. Interestingly we observed a dramatic statistically significant decrease in CD3+ T cells at day 3 in the intestinal epithelium. This may be explained with the ability of the pathogen to downregulate the expansion of newly recruited T cells into the epithelium, impairing the immune response. At day 3, B lymphocytes were absent in both C and I groups in the epithelium and they slightly increased in the I group in *lamina propria*, as indicated by CD20 staining. On the contrary, at day 9, a statistically significant increase in CD3+ T cells was observed in the I group in the intestinal epithelium and a slight increase (not statistically significant) in the *lamina propria*, probably as result of the effective host immune response, triggered by the cytokines. No differences in B lymphocytes were observed at day 9 between C and I groups, either in the intestinal epithelium nor in *lamina propria*. Overall, these results indicate a predominant cellular immune response at intestinal level, both in acute and chronic phases, that can be considered for the in vivo evaluation of alternative to antibiotic compounds.

T cells play a major role in pathogenesis of *E. coli* diseases; they respond to antigens in a selective and balanced fashion that allows for mounting an effective cell-mediated immune response.

IgA, secreted by plasma cells in intestinal *lamina propria*, are a major component of the local immune barrier of the gut and play a crucial role in intestinal homeostasis and protection; therefore, they are used as indicator of intestinal mucosal immunity [39]. At day 3 post infection no statistical difference was observed between C and I groups, while at day 9, the intestinal scrapes of I pigs displayed a significant lower amount of IgA than C pigs as consequence of the bacterial infection [40]. It is unlikely that maternal IgA could be adsorbed by small intestinal enterocytes of few-week-old pigs due to intestinal closure and gut maturation [41]; in contrast, it is possible that maternal IgA interact with intestinal microflora in the lumen and subsequently regulate the host immune response. Since one of the roles of IgA in the intestine is to bind the pathogenic bacteria and promote the internalization of the complex by M cells, it is likely that the amount of IgA present in the intestinal lumen substantially decreases after bacterial invasion. In addition, IgA undergo strong conformational changes upon antigen engagement and binding, which may explain the lower amount of IgA measurable by immunoassays. This occurs in the intestine only, as confirmed by our data, which show that levels of IgA in serum are about the same in C vs. I groups at both considered time points. Thus, on one side, the intestinal barrier heavily affects the microorganism; on the other side, the infectious agent impairs the gut homeostasis by destabilizing intestinal mucosa.

The kinetic of serum IgA, similar in C and I groups, showed a decrease in titer from 3 to 9 days, (statistical tendency only for the C group) as expected in accordance with the normal decline in maternally derived antibodies in the post-weaning piglets [42].

The assessed panel of clinical symptoms can represent a useful tool in order to evaluate the effectiveness of a further intervention that mitigates or abate the severity of the *E. coli* O138 challenge. The clinical scores related to the acute symptoms (oedema, vitality, and depression) were discriminant after both 3 days and 9 days post-challenge and represent a consistent and valuable pattern of observation on a daily basis that can offer important information. On the contrary, the effect of the experimental infection on hair status was more indicative after 9 days post-challenge. Even if the observed inflammatory and immunity parameters provided interesting data, due to the high individual variability, a large sample numerosity could be necessary to better underline the differences.

## 5. Conclusions

The adopted infection model, carried out on susceptible piglets, allowed us to identify a panel of clinical symptoms related to VTEC infection that could be used to further assess the effectiveness of antibiotic alternatives. The specific molecular mechanisms by which intestinal bacteria induce host immune system alterations are still largely unknown, as it is unclear how the host immune response impacts the outcome of VTEC diseases. Nevertheless, our work contributes to uncover some immune traits of *E. coli* O138 infection in post-weaned piglets, e.g., how diet, host signalling immune molecules and microorganism interplay and allows us to identify some fundamental parameters of the host immune response to be taken in consideration in further studies.

## Figures and Tables

**Table 1:** Composition of the experimental diet.

Ingredient, g/kg	Basal Diet	High Protein Diet
Barley	228.000	161.880
Wheat flakes	168.000	119.280
Wheat meal	134.000	95.140
Maize flakes	112.000	79.520
Barley flakes	72.600	51.546
Soy protein concentrate	70.000	49.700
Soybean meal	-	28.000
Whey	55.600	39.476
Maize meal	52.000	36.920
Fish meal (herring)	39.700	28.187
Monohydrate dextrose	37.100	26.341
Spray-dried plasma	27.100	19.241
Coconut oil	27.000	19.170
Soybean oil	16.000	11.360
Dicalcium phosphate	5.200	3.692
Calcium carbonate	1.100	0.781
Sodium butyrate 30% ^1^	2.190	1.555
L-Lys	6.700	4.757
DL-Met	3.220	2.286
L-Thr	3.040	2.158
L-Trp	1.220	0.866
Vitamin/mineral premix ^2^	3.270	2.322
Vitamin E 50%	0.140	0.099
Additives: phytase ^3^, xylanase ^4^, Acidifiers ^5^, feed flavours	16.550	11.750
Calculated Composition		
Dry matter	90.33	88.50
CP	17.80	25.42
EE	5.87	4.57
CF	2.19	3.20
Ashes	4.46	5.17
Starch + sugar	51.51	38.88
Lysine	1.48	1.73
NE Mcal/kg	2.60	3.56

Ingredient and chemical composition of the experimental diets (g/kg, as-fed basis). ^1^ Palm oil, salts of fatty acids (sodium butyrate 30%), calcium carbonate. ^2^ Providing the following nutrients (per kg of air-dried diet): Vitamin A, (E 672) 4,000,000 UI/kg; Vitamin D3, (E 671) 400,000 UI/kg; Vitamin E, (3a700) 40,000 mg/kg; Vitamin B1, 1200 mg/kg; Vitamin B2, 4000 mg/kg; Calcium D-pantothenate, (3a841) 10,865 mg/kg; Vitamin B6, (3a831) 2400 mg/kg; Vitamin B12, 16 mg/kg; niacinamide, (3a315) 14,000 mg/kg; Vitamin K3, 2000 mg/kg; Folic acid, (3a316) 600 mg/kg; D-Biotin, 80 mg/kg; Choline chloride, (3a890) 90,000 mg/kg, Fe (FeO), 45,000 mg/kg; Cu (CuSO4), 8000 mg/kg; Zn (ZnO), 52,200 mg/kg; Mn (MnO), 16,000 mg/kg; I (Ca(IO3)2), 240 mg/kg; Se (Na2SeO3), 120 mg/kg. ^3^ Phytase (EC 3.1.3.26) minimum: 10,000 Fytα-phytase/g. ^4^ Endo-1,4β-Xylanase (IUB/EC 3.2.1.8) minimum 1000 FXU/g. ^5^ Ortophosphoric acid 33.5%, Calcium formate 32.38%, Citric acid 7.8%, Fumaric Acid 5%, Silicic acid 6%. CP: crude protein; EE: ether extract; CF: crude fibre.

**Table 2:** Oligonucleotides used for PCR reactions.

Gene	Nucleotide Sequence (5′ to 3′)	Accession Number (GenBank)
TLR-2, F	GACACCGCCATCCTCATTCT	GU138028
TLR-2, R	CTTCCCGCTGCGTCTCAT	
TLR-4, F	GCCTTTCTCTCCTGCCTGAG	AB188301
TLR-4, R	AGCTCCATGCATTGGTAACTAATG	
IFN-γ, F	GCCAGGCGCCCTTTTTTA	NM_213948
IFN- γ, R	CTCTCCTCTTTCCAATTCTTCAAAAT	
IL1-β, F	ACGGTGACAACAATAATGACCTGT	NM_214055
IL1-β, R	CAAGGTCCAGGTTTTGGGTG	
MHC-I, F	CGCACAGACTTTCCGAGTG	AF464005
MHC-I, R	GTCTGGTCCCAAGTAGCAG	
MHC-II, F	CAAGCACTGGGAGTTTGAAG	DQ883222
MHC-II, R	ACACCCTTGATGATGAGGAC	
β-actin, F	CTCCTTCCTGGGCATGGAG	DQ845171
β-actin, R	GAGTTGAAGGTGGTCTCGTGG	

**Table 3:** Total median scores related to clinical symptoms.

	Controls			Infected			Significance
	Median	Min	Max	Median	Min	Max	*p > t*
Epiphora_Σ3	1.00	1.00	3.00	2.50	1.00	6.00	0.0342
Epiphora_Σ9	5.00	3.00	10.00	8.00	3.00	14.00	0.3031
Oedema_Σ3	1.00	0.00	2.00	2.00	1.00	3.00	00345
Oedema_Σ9	3.00	2.00	5.00	9.00	7.00	14.00	0.0061
Vitality_Σ3	0.00	0.00	0.00	2.00	1.00	4.00	0.0004
Vitality_Σ9	0.00	0.00	0.00	2.00	1.00	14.00	0.0048
Depression_Σ3	0.00	0.00	1.00	1.00	0.00	3.00	0.0099
Depression_Σ9	0.00	0.00	1.00	5.00	2.00	12.00	0.0052
Hair_Σ3	1.50	1.00	2.00	2.00	0.00	4.00	0.5432
Hair_Σ9	7.00	5.00	8.00	10.50	7.00	20.00	0.0203
Perineal area_Σ3	0.50	0.00	2.00	1.00	0.00	3.00	0.5774
Perineal area_Σ9	0.50	0.00	6.00	3.50	0.00	9.00	0.2279
Faecal score_Σ3	4.00	3.00	7.00	6.00	1.00	9.00	0.2764
Faecal score_Σ9	10.00	5.00	11.00	11.50	5.00	25.00	0.3891

Note: Σ3: sum of the individual daily scores from days 1 to 3 (*n* = 18); Σ9: sum of the individual daily scores from days 1 to 9 (*n* = 12). The comparisons were performed using the Wilcoxon Rank Sum test for independent groups.

**Table 4:** Median parameters obtained in infected and controls at 3 and 9 days.

Time	Method	Parameter	Controls			Infected			Significance
			Median	Min	Max	Median	Min	Max	*p > t*
3 days post-infection	Histology	Infiltrates of lymphocytes	2.00	2.00	2.00	2.00	1.00	3.00	1.0000
		Epithelial regeneration	0.50	0.00	1.00	0.00	0.00	1.00	0.5762
		Fusion of villi	0.50	0.00	2.00	1.00	0.00	2.00	0.5428
		Oedema	0.50	0.00	1.00	0.00	0.00	1.00	0.5762
		T-Atrophy	0.50	0.00	1.00	1.00	0.00	1.00	0.5762
		Stroma	0.50	0.00	1.00	0.50	0.00	1.00	1.0000
		Follicular hyperplasia	0.00	0.00	0.00	0.50	0.00	1.00	0.2636
	Immunohistochemistry	CD3 in epithelium	4.00	4.00	4.00	3.00	2.00	3.00	0.0455
		CD3 in *lamina propria*	4.00	4.00	4.00	3.50	3.00	4.00	0.2636
		CD20 in epithelium	0.00	0.00	0.00	0.00	0.00	0.00	1.0000
		CD20 in *lamina propria*	1.00	1.00	1.00	1.00	1.00	2.00	0.4795
		lba1 in villus	4.00	4.00	4.00	4.00	3.00	4.00	0.4795
		lb1 in crypts	2.00	2.00	2.00	2.00	2.00	3.00	0.4795
		IgG in luminal surface	1.00	0.00	2.00	0.50	0.00	2.00	0.8026
		IgG in villus axis	1.00	1.00	1.00	1.00	1.00	2.00	0.4795
		IgG in crypts	2.50	2.00	3.00	2.00	1.00	2.00	0.1709
		IgA-S luminal surface	0.50	0.00	1.00	0.50	0.00	1.00	1.0000
		IgA in villus axis	0.50	0.00	1.00	0.00	0.00	0.00	0.1573
		IgA in crypts	1.00	0.00	2.00	2.00	1.00	2.00	0.4113
9 days post-infection	Histology	Infiltrates of lymphocytes	1.00	1.00	1.00	2.00	1.00	3.00	0.0102
		Epithelial regeneration	0.00	0.00	1.00	1.00	0.00	2.00	0.2453
		Fusion of villi	0.00	0.00	1.00	1.00	0.00	2.00	0.1670
		Oedema	0.00	0.00	1.00	0.00	0.00	1.00	1.0000
		T-Atrophy	0.50	0.00	2.00	0.00	0.00	1.00	0.3074
		Stroma	1.50	0.00	2.00	0.00	0.00	2.00	0.1670
		Follicular hyperplasia	0.00	0.00	0.00	0.00	0.00	0.00	1.0000
	Immunohistochemistry	CD3 in epithelium	2.50	2.00	3.00	3.50	3.00	4.00	0.0261
		CD3 in *lamina propria*	3.00	3.00	3.00	3.00	2.00	4.00	0.6547
		CD20 in epithelium	0.00	0.00	0.00	0.00	0.00	0.00	1.0000
		CD20 in *lamina propria*	1.00	1.00	1.00	1.00	1.00	1.00	1.0000
		lba1 in villus	4.00	3.00	4.00	4.00	3.00	4.00	0.6000
		lb1 in crypts	2.00	2.00	3.00	2.00	2.00	3.00	0.6785
		IgG in luminal surface	0.50	0.00	2.00	0.00	0.00	1.00	0.3074
		IgG in villus axis	1.00	1.00	1.00	1.00	1.00	2.00	0.4795
		IgG in crypts	2.00	2.00	3.00	2.50	2.00	3.00	0.4279
		IgA-S luminal surface	0.00	0.00	2.00	0.00	0.00	1.00	0.9187
		IgA in villus axis	0.00	0.00	0.00	0.00	0.00	0.00	1.0000
		IgA in crypts	2.00	1.00	2.00	2.00	2.00	3.00	0.1175

Note: Infiltrates of lymphocytes, epithelial regeneration, fusion of villi, oedema, T-Atrophy, stroma and follicular hyperplasia were semi-quantitatively scored (0 = absent, 1 = slight, 2 = moderate; 3 = strong). CD3, CD20 and Iba1 immunoreactions were semi-quantitatively scored in *lamina propria* (0 = absent, 1 = rare cells, 2 = some cells; 3 = numerous cells; 4 = very numerous cells). For IgG and IgA, immunoreaction was semi-quantitatively scored on the epithelial luminal surface and *lamina propria* (0 = absent, 1 = slight, 2 = moderate; 3 = strong). The comparisons were performed using the Wilcoxon Rank Sum test for independent groups.

**Table 5:** Median histology and immunohistochemistry parameters obtained at 3 and 9 days for infected and controls.

Group	Method	Parameter	Day 3			Day 9			Significance
			Median	Min	Max	Median	Min	Max	*p > t*
Infected	Histology	Infiltrates of lymphocytes	2.00	1.00	3.00	2.00	1.00	3.00	0.7739
		Epithelial regeneration	0.00	0.00	1.00	1.00	0.00	2.00	0.2453
		Fusion of villi	1.00	0.00	2.00	1.00	0.00	2.00	1.0000
		Oedema	0.00	0.00	1.00	0.00	0.00	1.00	1.0000
		T-Atrophy	1.00	0.00	1.00	0.00	0.00	1.00	0.1128
		Stroma	0.50	0.00	1.00	0.00	0.00	2.00	0.8465
		Follicular hyperplasia	0.50	0.00	1.00	0.00	0.00	0.00	0.0359
	Immunohistochemistry	CD3 in epithelium	3.00	2.00	3.00	3.50	3.00	4.00	0.0528
		CD3 in *lamina propria*	3.50	3.00	4.00	3.00	2.00	4.00	0.3329
		CD20 in epithelium	0.00	0.00	0.00	0.00	0.00	0.00	1.0000
		CD20 in *lamina propria*	1.00	1.00	2.00	1.00	1.00	1.00	0.1573
		lba1 in villus	4.00	3.00	4.00	4.00	3.00	4.00	0.6000
		lb1 in crypts	2.00	2.00	3.00	2.00	2.00	3.00	0.6785
		IgG in luminal surface	0.50	0.00	2.00	0.00	0.00	1.00	0.3074
		IgG in villus axis	1.00	1.00	2.00	1.00	1.00	2.00	0.6000
		IgG in crypts	2.00	1.00	2.00	2.50	2.00	3.00	0.0528
		IgA-S luminal surface	0.50	0.00	1.00	0.00	0.00	1.00	0.6918
		IgA in villus axis	0.00	0.00	0.00	0.00	0.00	0.00	1.0000
		IgA in crypts	2.00	1.00	2.00	2.00	2.00	3.00	0.1175
Controls	Histology	Infiltrates of lymphocytes	2.00	2.00	2.00	1.00	1.00	1.00	0.0253
		Epithelial regeneration	0.50	0.00	1.00	0.00	0.00	1.00	0.5762
		Fusion of villi	0.50	0.00	2.00	0.00	0.00	1.00	0.5762
		Oedema	0.50	0.00	1.00	0.00	0.00	1.00	0.5762
		T-Atrophy	0.50	0.00	1.00	0.50	0.00	2.00	0.8026
		Stroma	0.50	0.00	1.00	1.50	0.00	2.00	0.3329
		Follicular hyperplasia	0.00	0.00	0.00	0.00	0.00	0.00	1.0000
	Immunohistochemistry	CD3 in epithelium	4.00	4.00	4.00	2.50	2.00	3.00	0.0528
		CD3 in *lamina propria*	4.00	4.00	4.00	3.00	3.00	3.00	0.0253
		CD20 in epithelium	0.00	0.00	0.00	0.00	0.00	0.00	1.0000
		CD20 in *lamina propria*	1.00	1.00	1.00	1.00	1.00	1.00	1.0000
		lba1 in villus	4.00	4.00	4.00	4.00	3.00	4.00	0.4795
		lb1 in crypts	2.00	2.00	2.00	2.00	2.00	3.00	0.4795
		IgG in luminal surface	1.00	0.00	2.00	0.50	0.00	2.00	0.8026
		IgG in villus axis	1.00	1.00	1.00	1.00	1.00	1.00	1.0000
		IgG in crypts	2.50	2.00	3.00	2.00	2.00	3.00	0.5762
		IgA-S luminal surface	0.50	0.00	1.00	0.00	0.00	2.00	0.7842
		IgA in villus axis	0.50	0.00	1.00	0.00	0.00	0.00	0.1573
		IgA in crypts	1.00	0.00	2.00	2.00	1.00	2.00	0.4113

Note: Infiltrates of lymphocytes, epithelial regeneration, fusion of villi, oedema, T-Atrophy, stroma and follicular hyperplasia were semi-quantitatively scored (0 = absent, 1 = slight, 2 = moderate; 3 = strong). CD3, CD20 and Iba1 immunoreactions were semi-quantitatively scored in *lamina propria* (0 = absent, 1 = rare cells, 2 = some cells; 3 = numerous cells; 4 = very numerous cells). For IgG and IgA, immunoreaction was semi-quantitatively scored on the epithelial luminal surface and *lamina propria* (0 = absent, 1 = slight, 2 = moderate; 3 = strong). The comparisons were performed using the Wilcoxon Rank Sum test for independent groups.

**Table 6:** Median PCR and ELISA parameters obtained at 3 and 9 days for infected and controls.

Time	Method	Parameter	Day 3			Day 9			Significance
			Median	Min	Max	Median	Min	Max	*p > t*
Infected	Real-Time PCR ^1^	MHC-I (lymph nodes)	42.34	12.86	42.34	65.79	0.07	176.07	0.4969
		MHC-II (lymph nodes)	0.46	0.25	0.66	0.735	0.01	1.31	0.0894
		IFN-γ (jejunum)	6.80	0.03	213.04	0.89	0.10	36.89	0.3082
		IL-1β (jejunum)	0.51	0.45	0.81	0.63	0.17	1.23	0.7336
		TLR2 (jejunum)	1.19	0.25	15.94	0.53	0.11	2.58	0.2345
		TLR4 (jejunum)	0.51	0.48	2.35	0.49	0.17	1.62	0.4439
	ELISA	IgA serum (mg/mL)	0.51	0.12	0.57	0.19	0.10	0.70	0.3436
		IgA scrape (µg/mL)	99.44	98.32	156.01	98.16	96.83	104.22	0.3949
		CXC9L scrape (ng/mg TP)	0.96	0.15	2.88	1.98	0.66	4.55	0.1742
		TNF-α scrape (ng/mg TP)	0.37	0.07	1.62	0.72	0.35	1.15	0.3494
		IL_8 scrape (ng/mg TP)	3.33	0.25	4.55	3.42	2.11	5.19	0.7341
		IL-1 scrape (pg/mL)	0.07	0.01	0.21	0.07	0.02	0.12	0.8649
Controls	Real-Time PCR ^1^	MHC-I (lymph nodes)	13.27	13.04	13.50	5.80	0.23	37.40	0.3545
		MHC-II (lymph nodes)	0.65	0.51	0.78	0.72	0.00	1.62	1.0000
		IFN-γ (jejunum)	11.42	1.36	21.48	0.47	0.04	4.27	0.1649
		IL-1β (jejunum)	0.57	0.12	1.01	0.53	0.37	0.76	1.0000
		TLR2 (jejunum)	0.78	0.58	0.98	0.40	0.36	0.79	0.1649
		TLR4 (jejunum)	1.09	0.36	1.82	0.41	0.38	0.51	1.0000
	ELISA	IgA serum (mg/mL)	0.57	0.54	0.59	0.23	0.16	0.39	0.0641
		IgA scrape (µg/mL)	98.94	96.67	101.20	105.49	99.89	112.95	0.3545
		CXC9L scrape (ng/mg TP)	1.10	0.98	1.22	2.20	1.56	2.84	0.0641
		TNF-α scrape (ng/mg TP)	0.34	0.31	0.37	0.81	0.57	0.98	0.0641
		IL_8 scrape (ng/mg TP)	1.74	1.69	1.79	3.22	1.49	4.31	0.3545
		IL-1 scrape (pg/mL)	0.06	0.06	0.06	0.11	0.06	0.16	0.1336

^1^ Data were normalized to beta-actin expression and reported as relative expression. TP: Total protein. The comparisons were performed using the Wilcoxon Rank Sum test for independent groups.

**Table 7:** Median parameters obtained in infected and controls at 3 and 9 days.

Time	Method	Parameter	Controls			Infected			Significance
			Median	Min	Max	Median	Min	Max	*p > t*
3 days post-infection	Real-Time PCR ^1^	MHC-I (lymph nodes)	13.27	13.04	13.50	42.34	12.86	42.34	0.3545
		MHC-II (lymph nodes)	0.65	0.51	0.78	0.46	0.25	0.66	0.1649
		IFN-γ (jejunum)	11.42	1.36	21.48	6.80	0.03	213.04	1.0000
		IL-1β (jejunum)	0.57	0.12	1.01	0.51	0.45	0.81	1.0000
		TLR2 (jejunum)	0.78	0.58	0.98	1.19	0.25	15.94	0.6434
		TLR4 (jejunum)	1.09	0.36	1.82	0.51	0.48	2.35	0.6434
	ELISA	IgA serum (mg/mL)	0.57	0.54	0.59	0.51	0.12	0.57	0.1649
		IgA scrape (µg/mL)	98.94	96.67	101.20	99.44	98.32	156.01	0.6386
		CXC9L scrape (ng/mg TP)	1.10	0.98	1.22	0.96	0.15	2.88	1.0000
		TNF-α scrape (ng/mg TP)	0.34	0.31	0.37	0.37	0.07	1.62	1.0000
		IL_8 scrape (ng/mg TP)	1.74	1.69	1.79	3.33	0.25	4.55	0.3545
		IL-1 scrape (pg/mL)	0.06	0.06	0.06	0.07	0.01	0.21	1.0000
9 days post-infection	Real-Time PCR ^1^	MHC-I (lymph nodes)	5.80	0.23	37.40	65.79	0.07	176.07	0.1742
		MHC-II (lymph nodes)	0.72	0.00	1.62	0.735	0.01	1.31	1.0000
		IFN-γ (jejunum)	0.47	0.04	4.27	0.89	0.10	36.89	0.2345
		IL-1β (jejunum)	0.53	0.37	0.76	0.63	0.17	1.23	0.8649
		TLR2 (jejunum)	0.40	0.36	0.79	0.53	0.11	2.58	0.7341
		TLR4 (jejunum)	0.41	0.38	0.51	0.49	0.17	1.62	0.3949
	ELISA	IgA serum (mg/mL)	0.23	0.16	0.39	0.19	0.10	0.70	0.7048
		IgA scrape (µg/mL)	105.49	99.89	112.95	98.16	96.83	104.22	0.0740
		CXC9L scrape (ng/mg TP)	2.20	1.56	2.84	1.98	0.66	4.55	1.0000
		TNF-α scrape (ng/mg TP)	0.81	0.57	0.98	0.72	0.35	1.15	0.8651
		IL_8 scrape (ng/mg TP)	3.22	1.49	4.31	3.42	2.11	5.19	0.6104
		IL-1 scrape (pg/mL)	0.11	0.06	0.16	0.07	0.02	0.12	0.1980

^1^ Data were normalized to beta-actin expression and reported as relative expression. TP: Total protein. The comparisons were performed using the Wilcoxon Rank Sum test for independent groups.

## Data Availability

All data are available within the article.
